# [*N*′-(5-Chloro-2-oxidobenzyl-κ*O*)-2,4-dihy­droxy­benzohydrazidato-κ^2^ *N*′,*O*](methanol-κ*O*)dioxidomolybdenum(VI)–4,4′-bipyridine (1/1)

**DOI:** 10.1107/S1600536811017259

**Published:** 2011-05-14

**Authors:** Ngui Khiong Ngan, Richard Chee Seng Wong, Kong Mun Lo, Seik Weng Ng

**Affiliations:** a Department of Chemistry, University of Malaya, 50603 Kuala Lumpur, Malaysia

## Abstract

In the title co-crystal, [Mo(C_14_H_9_ClN_2_O_4_)O_2_(CH_3_OH)]·C_10_H_8_N_2_, the deprotonated Schiff base *O*,*N*,*O*′-chelates to the Mo^VI^ atom, the three atoms involved in chelation comprising the *fac* sites of the octa­hedron surrounding the methanol-coordinated metal atom. The methanol mol­ecule forms an O—H⋯N hydrogen bond to an N atom of the 4,4′-bipyridine solvent mol­ecule; the hy­droxy group of the Schiff base forms an O—H⋯N hydrogen bond to the other N atom of another mol­ecule. The two hydrogen bonds leading to the formation of a helical chain running along the *b* axis.

## Related literature

For a related Mo^VI^O_2_–4′,4-bipyridine adduct, see: Dinda *et al.* (2006 ▶). For the structure of the unsubstituted Schiff base, see: Pan & Yang (2005 ▶).
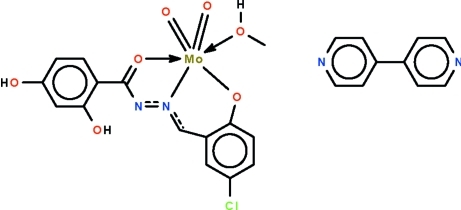


## Experimental

### 

#### Crystal data

[Mo(C_14_H_9_ClN_2_O_4_)O_2_(CH_4_O)]·C_10_H_8_N_2_
*M* *_r_* = 620.85
Monoclinic, 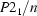

*a* = 6.9575 (3) Å
*b* = 7.4541 (4) Å
*c* = 47.197 (2) Å
β = 92.0073 (6)°
*V* = 2446.2 (2) Å^3^
*Z* = 4
Mo *K*α radiation
μ = 0.70 mm^−1^
*T* = 100 K
0.25 × 0.20 × 0.20 mm

#### Data collection

Bruker SMART APEX diffractometer
Absorption correction: multi-scan (*SADABS*; Sheldrick, 1996 ▶) *T*_min_ = 0.885, *T*_max_ = 1.000
29717 measured reflections
5604 independent reflections
5408 reflections with *I* > 2σ(*I*)
*R*_int_ = 0.032

#### Refinement

*R*[*F* ^2^ > 2σ(*F* ^2^)] = 0.049
*wR*(*F* ^2^) = 0.138
*S* = 1.23
5604 reflections
356 parameters
3 restraints
H atoms treated by a mixture of independent and constrained refinement
Δρ_max_ = 0.55 e Å^−3^
Δρ_min_ = −1.25 e Å^−3^

### 

Data collection: *APEX2* (Bruker, 2009 ▶); cell refinement: *SAINT* (Bruker, 2009 ▶); data reduction: *SAINT*; program(s) used to solve structure: *SHELXS97* (Sheldrick, 2008 ▶); program(s) used to refine structure: *SHELXL97* (Sheldrick, 2008 ▶); molecular graphics: *X-SEED* (Barbour, 2001 ▶); software used to prepare material for publication: *publCIF* (Westrip, 2010 ▶).

## Supplementary Material

Crystal structure: contains datablocks global, I. DOI: 10.1107/S1600536811017259/jh2289sup1.cif

Structure factors: contains datablocks I. DOI: 10.1107/S1600536811017259/jh2289Isup2.hkl

Additional supplementary materials:  crystallographic information; 3D view; checkCIF report

## Figures and Tables

**Table 1:** Hydrogen-bond geometry (Å, °)

*D*—H⋯*A*	*D*—H	H⋯*A*	*D*⋯*A*	*D*—H⋯*A*
O2—H2⋯N1	0.84 (1)	1.85 (3)	2.600 (4)	147 (6)
O3—H3⋯N3	0.84 (1)	1.92 (2)	2.741 (5)	166 (6)
O7—H7⋯N4^i^	0.84 (1)	1.84 (1)	2.679 (4)	175 (6)

Symmetry code: (i) 
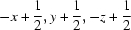
.

**Fig. 1.**
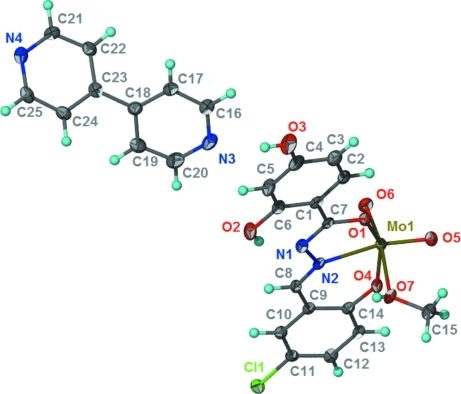
Thermal ellipsoid plot (Barbour, 2001) of MoO2(CH3OH)(C14H9ClN2O4).C10H8N2 at the 70% probability level; hydrogen atoms are drawn as spheres of arbitrary radius.

## supplementary crystallographic information

### Comment

The Schiff bases that are synthesized by condensing salicylaldehyde (and its substituted analogs) with benzohydrazide (and its substituted analogs) function as terdentate *O*,*N*,*O*'-chelates to a wide range of metal ions. A large number of metal derivatives have been reported; in octahedral systems, the ligand generally exists as a doubly-deprotonated species that chelates in a *fac* manner. The crystal structure of the parent (unsubstituted) ligand has been reported (Pan & Yang, 2005); a dioxomolybdenum(VI) derivative is known in which 4,4'-bipyridine binds to two metal atoms (Dinda *et al.*, 2006). In the present study, using a Schiff base that possesses substitutents leads to a solvent-coordinated derivative in which 4,4'-bipyridine interacts indirectly, through the solvent molecule, in an outer-sphere coordination mode. In the co-crystal, MoO_2_(CH_3_OH)(C_14_H_9_ClN_2_O_4_^).^C_10_H_8_N_2_(Scheme I), the deprotonated Schiff base *O*,*N*,*O*'-chelates to the Mo^VI^ atom, the three atoms involved in chelation comprising the *fac* sites of the octahedron surrounding the methanol-coordinated metal center (Fig. 1). The solvent molecule forms an O–H···N hydrogen bond to a N atom of the 4,4'-bipyridine molecule; the hydroxy group forms an O–H···N hydrogen bond to the other N atom of another molecule, the two hydrogen bonds leading to the formation of a helical chain that runs along the *b*-axis of the monoclinic unit cell (Table 1).

### Experimental

3-Ethoxysalicylaldehyde (0.166 g, 1 mmol) and 2,4-dihydroxybenzohydrazide (0.156 g, 1 mmol) were condensed in methanol (100 ml). The solution was heated to give a yellow coloration. The cool solution yielded the desired Schiff base as a yellow compound. The ligand (0.306 g, 1 mmol) and di(acetylacetonato)dioxomolybdenum(VI) (0.328 g, 1 mmol) were dissolved in heated in methanol for an hour. To the orange-red solution was added 4,4'-bipyridine (0.08 g, 0.5 mmol); heating was continued for another hour. The solution was filtered and set aside for the growth of crystals, m.p. 533–535 K.

### Refinement

Carbon-bound H-atoms were placed in calculated positions (C—H 0.95 to 0.99 Å) and were included in the refinement in the riding model approximation, with *U*(H) set to 1.2 times *U*_eq_(C). The oxygen-bound H-atoms were located in a difference Fourier map and were refined with a distance restraint of O–H 0.84±0.01 Å; their temperature factors were freely refined.

The final difference Fourier map had a hole in the vicinity of O6.

Omitted because of bad disagreements were the (0 0 2) and (0 0 4) reflections.

The second parameter in the weighting scheme is somewhat large; lowering this had only a marginal effect on the refinement.

### Crystal data

**Table:**

[Mo(C_14_H_9_ClN_2_O_4_)O_2_(CH_4_O)]·C_10_H_8_N_2_	*F*(000) = 1256
*M**_r_* = 620.85	*D*_x_ = 1.686 Mg m^−^^3^
Monoclinic, *P*2_1_/*n*	Mo *K*α radiation, λ = 0.71073 Å
Hall symbol: -P 2yn	Cell parameters from 9874 reflections
*a* = 6.9575 (3) Å	θ = 2.6–28.2°
*b* = 7.4541 (4) Å	µ = 0.70 mm^−^^1^
*c* = 47.197 (2) Å	*T* = 100 K
β = 92.0073 (6)°	Prism, orange–red
*V* = 2446.2 (2) Å^3^	0.25 × 0.20 × 0.20 mm
*Z* = 4	

### Data collection

**Table:**

Bruker SMART APEX diffractometer	5604 independent reflections
Radiation source: fine-focus sealed tube	5408 reflections with *I* > 2σ(*I*)
graphite	*R*_int_ = 0.032
ω scans	θ_max_ = 27.5°, θ_min_ = 2.6°
Absorption correction: multi-scan (*SADABS*; Sheldrick, 1996)	*h* = −9→9
*T*_min_ = 0.885, *T*_max_ = 1.000	*k* = −9→9
29717 measured reflections	*l* = −61→61

### Refinement

**Table:**

Refinement on *F*^2^	Primary atom site location: structure-invariant direct methods
Least-squares matrix: full	Secondary atom site location: difference Fourier map
*R*[*F*^2^ > 2σ(*F*^2^)] = 0.049	Hydrogen site location: inferred from neighbouring sites
*wR*(*F*^2^) = 0.138	H atoms treated by a mixture of independent and constrained refinement
*S* = 1.23	*w* = 1/[σ^2^(*F*_o_^2^) + (0.058*P*)^2^ + 9.6979*P*] where *P* = (*F*_o_^2^ + 2*F*_c_^2^)/3
5604 reflections	(Δ/σ)_max_ = 0.001
356 parameters	Δρ_max_ = 0.55 e Å^−^^3^
3 restraints	Δρ_min_ = −1.25 e Å^−^^3^

### Fractional atomic coordinates and isotropic or equivalent isotropic displacement parameters (Å^2^)

**Table:**

	*x*	*y*	*z*	*U*_iso_*/*U*_eq_	
Mo1	1.01769 (4)	0.07720 (4)	0.416314 (7)	0.01254 (11)	
Cl1	0.25401 (13)	−0.34030 (13)	0.50086 (2)	0.0185 (2)	
O1	0.9736 (4)	0.1896 (4)	0.37790 (6)	0.0160 (5)	
O2	0.4441 (4)	0.1634 (4)	0.33108 (6)	0.0213 (6)	
H2	0.471 (8)	0.126 (8)	0.3475 (6)	0.040 (17)*	
O3	0.7322 (5)	0.4673 (5)	0.25549 (6)	0.0303 (8)	
H3	0.624 (4)	0.454 (8)	0.2474 (11)	0.031 (15)*	
O4	0.9260 (4)	0.0010 (4)	0.45224 (6)	0.0156 (5)	
O5	1.2262 (4)	0.1843 (4)	0.42621 (6)	0.0173 (6)	
O6	1.0877 (4)	−0.1250 (4)	0.40328 (6)	0.0191 (6)	
O7	0.8759 (4)	0.3422 (4)	0.42838 (6)	0.0161 (5)	
H7	0.804 (6)	0.394 (7)	0.4163 (9)	0.029 (14)*	
N1	0.6590 (5)	0.0904 (4)	0.37578 (7)	0.0149 (6)	
N2	0.7088 (5)	0.0358 (4)	0.40308 (6)	0.0126 (6)	
N3	0.4092 (5)	0.3848 (5)	0.22321 (7)	0.0218 (7)	
N4	−0.1397 (5)	0.0211 (5)	0.10765 (7)	0.0187 (7)	
C1	0.7832 (6)	0.2412 (5)	0.33577 (8)	0.0151 (7)	
C2	0.9404 (6)	0.3226 (5)	0.32289 (8)	0.0177 (8)	
H2A	1.0618	0.3255	0.3327	0.021*	
C3	0.9220 (7)	0.3981 (6)	0.29631 (9)	0.0232 (9)	
H3A	1.0287	0.4556	0.2882	0.028*	
C4	0.7447 (6)	0.3896 (6)	0.28138 (8)	0.0215 (8)	
C5	0.5880 (6)	0.3080 (6)	0.29358 (9)	0.0221 (8)	
H5	0.4681	0.3025	0.2834	0.027*	
C6	0.6056 (6)	0.2349 (5)	0.32048 (8)	0.0166 (7)	
C7	0.8056 (5)	0.1687 (5)	0.36418 (8)	0.0139 (7)	
C8	0.5761 (5)	−0.0460 (5)	0.41652 (8)	0.0139 (7)	
H8	0.4564	−0.0673	0.4068	0.017*	
C9	0.5994 (5)	−0.1073 (5)	0.44556 (8)	0.0127 (7)	
C10	0.4417 (5)	−0.1914 (5)	0.45779 (8)	0.0151 (7)	
H10	0.3276	−0.2131	0.4467	0.018*	
C11	0.4521 (5)	−0.2421 (5)	0.48572 (8)	0.0144 (7)	
C12	0.6185 (6)	−0.2137 (5)	0.50248 (8)	0.0155 (7)	
H12	0.6237	−0.2496	0.5218	0.019*	
C13	0.7755 (5)	−0.1333 (5)	0.49074 (8)	0.0129 (7)	
H13	0.8895	−0.1148	0.5020	0.015*	
C14	0.7686 (5)	−0.0786 (5)	0.46234 (8)	0.0138 (7)	
C15	0.9788 (6)	0.4819 (6)	0.44335 (9)	0.0217 (8)	
H15A	0.8873	0.5691	0.4506	0.033*	
H15B	1.0655	0.5420	0.4305	0.033*	
H15C	1.0541	0.4298	0.4593	0.033*	
C16	0.4737 (6)	0.3877 (5)	0.19684 (8)	0.0183 (8)	
H16	0.5972	0.4377	0.1940	0.022*	
C17	0.3700 (6)	0.3215 (5)	0.17342 (8)	0.0172 (8)	
H17	0.4216	0.3272	0.1551	0.021*	
C18	0.1891 (6)	0.2466 (5)	0.17723 (9)	0.0179 (8)	
C19	0.1193 (6)	0.2480 (6)	0.20453 (9)	0.0233 (9)	
H19	−0.0055	0.2029	0.2080	0.028*	
C20	0.2334 (7)	0.3156 (7)	0.22641 (9)	0.0284 (10)	
H20	0.1846	0.3131	0.2450	0.034*	
C21	0.0510 (6)	0.0001 (6)	0.10986 (8)	0.0190 (8)	
H21	0.1123	−0.0677	0.0957	0.023*	
C22	0.1631 (6)	0.0724 (6)	0.13174 (9)	0.0190 (8)	
H22	0.2986	0.0558	0.1323	0.023*	
C23	0.0753 (6)	0.1701 (5)	0.15298 (8)	0.0165 (7)	
C24	−0.1230 (6)	0.1915 (5)	0.15073 (9)	0.0180 (8)	
H24	−0.1887	0.2568	0.1647	0.022*	
C25	−0.2235 (6)	0.1164 (6)	0.12788 (9)	0.0206 (8)	
H25	−0.3588	0.1332	0.1265	0.025*	

### Atomic displacement parameters (Å^2^)

**Table:**

	*U*^11^	*U*^22^	*U*^33^	*U*^12^	*U*^13^	*U*^23^
Mo1	0.01129 (17)	0.01290 (17)	0.01333 (17)	−0.00009 (11)	−0.00088 (11)	−0.00010 (12)
Cl1	0.0184 (4)	0.0176 (4)	0.0195 (4)	−0.0031 (3)	0.0036 (3)	0.0016 (4)
O1	0.0144 (12)	0.0193 (14)	0.0140 (13)	−0.0022 (10)	−0.0008 (10)	−0.0006 (11)
O2	0.0198 (14)	0.0291 (16)	0.0146 (14)	−0.0070 (12)	−0.0038 (11)	0.0040 (12)
O3	0.0366 (19)	0.042 (2)	0.0120 (14)	−0.0127 (16)	−0.0049 (13)	0.0059 (13)
O4	0.0149 (13)	0.0187 (14)	0.0130 (13)	−0.0028 (11)	−0.0029 (10)	0.0006 (11)
O5	0.0146 (13)	0.0192 (14)	0.0180 (13)	−0.0004 (11)	−0.0019 (10)	−0.0001 (11)
O6	0.0203 (14)	0.0187 (14)	0.0180 (14)	0.0025 (11)	−0.0017 (11)	−0.0023 (11)
O7	0.0202 (13)	0.0136 (13)	0.0140 (13)	0.0003 (11)	−0.0051 (10)	−0.0021 (10)
N1	0.0161 (15)	0.0154 (15)	0.0130 (15)	−0.0002 (12)	−0.0021 (12)	0.0018 (12)
N2	0.0162 (15)	0.0121 (14)	0.0093 (14)	0.0011 (12)	−0.0020 (11)	−0.0004 (11)
N3	0.0302 (19)	0.0229 (18)	0.0120 (16)	−0.0037 (15)	−0.0024 (14)	0.0007 (13)
N4	0.0209 (17)	0.0145 (16)	0.0205 (17)	−0.0039 (13)	−0.0019 (13)	0.0045 (13)
C1	0.0200 (18)	0.0128 (17)	0.0124 (17)	0.0009 (14)	−0.0010 (14)	−0.0027 (14)
C2	0.0204 (19)	0.0190 (19)	0.0136 (17)	−0.0053 (15)	0.0009 (14)	−0.0029 (15)
C3	0.028 (2)	0.025 (2)	0.0172 (19)	−0.0073 (17)	0.0025 (16)	−0.0024 (16)
C4	0.031 (2)	0.021 (2)	0.0124 (18)	−0.0050 (17)	0.0000 (16)	−0.0015 (15)
C5	0.024 (2)	0.026 (2)	0.0166 (19)	−0.0048 (17)	−0.0056 (15)	−0.0001 (16)
C6	0.0203 (19)	0.0148 (18)	0.0147 (17)	−0.0028 (14)	−0.0001 (14)	−0.0012 (14)
C7	0.0163 (17)	0.0133 (17)	0.0118 (16)	0.0011 (14)	−0.0015 (13)	−0.0050 (14)
C8	0.0127 (16)	0.0124 (17)	0.0165 (17)	−0.0002 (13)	−0.0013 (13)	−0.0011 (14)
C9	0.0159 (17)	0.0097 (16)	0.0124 (16)	0.0018 (13)	−0.0009 (13)	−0.0001 (13)
C10	0.0161 (17)	0.0112 (17)	0.0180 (18)	0.0014 (14)	−0.0001 (14)	−0.0014 (14)
C11	0.0159 (17)	0.0088 (16)	0.0186 (18)	−0.0005 (13)	0.0042 (14)	−0.0020 (14)
C12	0.0208 (19)	0.0085 (16)	0.0173 (18)	0.0023 (14)	−0.0007 (14)	0.0015 (14)
C13	0.0186 (17)	0.0103 (16)	0.0095 (16)	−0.0003 (14)	−0.0023 (13)	0.0020 (13)
C14	0.0170 (17)	0.0101 (16)	0.0143 (17)	0.0021 (14)	−0.0007 (14)	−0.0014 (14)
C15	0.025 (2)	0.0143 (18)	0.026 (2)	−0.0030 (16)	−0.0001 (16)	−0.0046 (16)
C16	0.024 (2)	0.0153 (18)	0.0159 (18)	−0.0008 (15)	−0.0006 (15)	0.0027 (15)
C17	0.0210 (19)	0.0188 (19)	0.0117 (17)	0.0020 (15)	0.0003 (14)	0.0018 (14)
C18	0.0179 (18)	0.0168 (18)	0.0191 (19)	0.0035 (15)	0.0008 (15)	−0.0021 (15)
C19	0.022 (2)	0.028 (2)	0.020 (2)	−0.0039 (17)	0.0056 (16)	0.0006 (17)
C20	0.034 (2)	0.037 (3)	0.0143 (19)	−0.006 (2)	0.0050 (17)	−0.0007 (18)
C21	0.024 (2)	0.0170 (19)	0.0164 (18)	0.0022 (16)	0.0019 (15)	−0.0018 (15)
C22	0.0150 (18)	0.0189 (19)	0.023 (2)	0.0009 (15)	0.0020 (15)	−0.0001 (16)
C23	0.0168 (18)	0.0149 (18)	0.0176 (18)	0.0025 (14)	−0.0016 (14)	0.0056 (15)
C24	0.0163 (18)	0.0144 (18)	0.024 (2)	0.0023 (14)	0.0044 (15)	0.0022 (15)
C25	0.0135 (18)	0.020 (2)	0.028 (2)	0.0010 (15)	−0.0011 (15)	0.0030 (17)

### Geometric parameters (Å, °)

**Table:**

Mo1—O6	1.705 (3)	C8—C9	1.448 (5)
Mo1—O5	1.707 (3)	C8—H8	0.9500
Mo1—O4	1.918 (3)	C9—C10	1.405 (5)
Mo1—O1	2.011 (3)	C9—C14	1.412 (5)
Mo1—N2	2.238 (3)	C10—C11	1.370 (5)
Mo1—O7	2.289 (3)	C10—H10	0.9500
Cl1—C11	1.737 (4)	C11—C12	1.395 (5)
O1—C7	1.326 (5)	C12—C13	1.379 (5)
O2—C6	1.355 (5)	C12—H12	0.9500
O2—H2	0.840 (10)	C13—C14	1.400 (5)
O3—C4	1.352 (5)	C13—H13	0.9500
O3—H3	0.840 (10)	C15—H15A	0.9800
O4—C14	1.347 (5)	C15—H15B	0.9800
O7—C15	1.436 (5)	C15—H15C	0.9800
O7—H7	0.838 (10)	C16—C17	1.389 (6)
N1—C7	1.311 (5)	C16—H16	0.9500
N1—N2	1.384 (4)	C17—C18	1.394 (6)
N2—C8	1.292 (5)	C17—H17	0.9500
N3—C16	1.338 (5)	C18—C19	1.393 (6)
N3—C20	1.341 (6)	C18—C23	1.483 (6)
N4—C21	1.337 (5)	C19—C20	1.376 (6)
N4—C25	1.340 (6)	C19—H19	0.9500
C1—C2	1.408 (5)	C20—H20	0.9500
C1—C6	1.410 (5)	C21—C22	1.382 (6)
C1—C7	1.449 (5)	C21—H21	0.9500
C2—C3	1.377 (6)	C22—C23	1.397 (6)
C2—H2A	0.9500	C22—H22	0.9500
C3—C4	1.401 (6)	C23—C24	1.389 (5)
C3—H3A	0.9500	C24—C25	1.383 (6)
C4—C5	1.390 (6)	C24—H24	0.9500
C5—C6	1.383 (6)	C25—H25	0.9500
C5—H5	0.9500		
			
O6—Mo1—O5	105.14 (14)	C10—C9—C8	117.8 (3)
O6—Mo1—O4	99.50 (13)	C14—C9—C8	123.1 (3)
O5—Mo1—O4	101.67 (12)	C11—C10—C9	120.2 (4)
O6—Mo1—O1	94.56 (13)	C11—C10—H10	119.9
O5—Mo1—O1	98.81 (12)	C9—C10—H10	119.9
O4—Mo1—O1	151.09 (11)	C10—C11—C12	121.2 (4)
O6—Mo1—N2	93.51 (13)	C10—C11—Cl1	119.7 (3)
O5—Mo1—N2	159.97 (13)	C12—C11—Cl1	119.0 (3)
O4—Mo1—N2	81.96 (11)	C13—C12—C11	119.4 (4)
O1—Mo1—N2	72.03 (11)	C13—C12—H12	120.3
O6—Mo1—O7	169.60 (12)	C11—C12—H12	120.3
O5—Mo1—O7	84.14 (12)	C12—C13—C14	120.7 (3)
O4—Mo1—O7	82.88 (11)	C12—C13—H13	119.7
O1—Mo1—O7	79.14 (11)	C14—C13—H13	119.7
N2—Mo1—O7	76.73 (11)	O4—C14—C13	117.8 (3)
C7—O1—Mo1	119.7 (2)	O4—C14—C9	122.7 (3)
C6—O2—H2	108 (4)	C13—C14—C9	119.5 (3)
C4—O3—H3	113 (4)	O7—C15—H15A	109.5
C14—O4—Mo1	138.0 (2)	O7—C15—H15B	109.5
C15—O7—Mo1	122.4 (2)	H15A—C15—H15B	109.5
C15—O7—H7	106 (4)	O7—C15—H15C	109.5
Mo1—O7—H7	119 (4)	H15A—C15—H15C	109.5
C7—N1—N2	110.3 (3)	H15B—C15—H15C	109.5
C8—N2—N1	115.9 (3)	N3—C16—C17	123.5 (4)
C8—N2—Mo1	128.6 (3)	N3—C16—H16	118.2
N1—N2—Mo1	115.2 (2)	C17—C16—H16	118.2
C16—N3—C20	116.6 (4)	C16—C17—C18	119.1 (4)
C21—N4—C25	117.5 (4)	C16—C17—H17	120.5
C2—C1—C6	118.3 (4)	C18—C17—H17	120.5
C2—C1—C7	120.0 (4)	C19—C18—C17	117.5 (4)
C6—C1—C7	121.7 (4)	C19—C18—C23	121.5 (4)
C3—C2—C1	121.4 (4)	C17—C18—C23	121.0 (4)
C3—C2—H2A	119.3	C20—C19—C18	119.1 (4)
C1—C2—H2A	119.3	C20—C19—H19	120.5
C2—C3—C4	119.5 (4)	C18—C19—H19	120.5
C2—C3—H3A	120.3	N3—C20—C19	124.1 (4)
C4—C3—H3A	120.3	N3—C20—H20	117.9
O3—C4—C5	122.3 (4)	C19—C20—H20	117.9
O3—C4—C3	117.6 (4)	N4—C21—C22	123.1 (4)
C5—C4—C3	120.1 (4)	N4—C21—H21	118.5
C6—C5—C4	120.5 (4)	C22—C21—H21	118.5
C6—C5—H5	119.8	C21—C22—C23	119.3 (4)
C4—C5—H5	119.8	C21—C22—H22	120.3
O2—C6—C5	116.5 (4)	C23—C22—H22	120.3
O2—C6—C1	123.2 (4)	C24—C23—C22	117.6 (4)
C5—C6—C1	120.3 (4)	C24—C23—C18	121.1 (4)
N1—C7—O1	122.3 (3)	C22—C23—C18	121.3 (4)
N1—C7—C1	119.5 (3)	C25—C24—C23	119.1 (4)
O1—C7—C1	118.2 (3)	C25—C24—H24	120.4
N2—C8—C9	123.7 (3)	C23—C24—H24	120.4
N2—C8—H8	118.1	N4—C25—C24	123.3 (4)
C9—C8—H8	118.1	N4—C25—H25	118.3
C10—C9—C14	119.0 (3)	C24—C25—H25	118.3
			
O6—Mo1—O1—C7	86.0 (3)	C2—C1—C7—N1	−178.8 (4)
O5—Mo1—O1—C7	−167.9 (3)	C6—C1—C7—N1	2.0 (6)
O4—Mo1—O1—C7	−33.2 (4)	C2—C1—C7—O1	3.3 (5)
N2—Mo1—O1—C7	−6.3 (3)	C6—C1—C7—O1	−175.8 (3)
O7—Mo1—O1—C7	−85.7 (3)	N1—N2—C8—C9	−177.8 (3)
O6—Mo1—O4—C14	−78.0 (4)	Mo1—N2—C8—C9	9.0 (5)
O5—Mo1—O4—C14	174.3 (4)	N2—C8—C9—C10	178.8 (4)
O1—Mo1—O4—C14	40.0 (5)	N2—C8—C9—C14	1.9 (6)
N2—Mo1—O4—C14	14.3 (4)	C14—C9—C10—C11	0.9 (5)
O7—Mo1—O4—C14	91.8 (4)	C8—C9—C10—C11	−176.1 (3)
O6—Mo1—O7—C15	−164.6 (6)	C9—C10—C11—C12	−0.7 (6)
O5—Mo1—O7—C15	−11.1 (3)	C9—C10—C11—Cl1	178.2 (3)
O4—Mo1—O7—C15	91.5 (3)	C10—C11—C12—C13	0.0 (6)
O1—Mo1—O7—C15	−111.2 (3)	Cl1—C11—C12—C13	−179.0 (3)
N2—Mo1—O7—C15	174.9 (3)	C11—C12—C13—C14	0.6 (6)
C7—N1—N2—C8	−178.9 (3)	Mo1—O4—C14—C13	170.9 (3)
C7—N1—N2—Mo1	−4.8 (4)	Mo1—O4—C14—C9	−9.5 (6)
O6—Mo1—N2—C8	85.6 (3)	C12—C13—C14—O4	179.3 (3)
O5—Mo1—N2—C8	−115.6 (4)	C12—C13—C14—C9	−0.4 (6)
O4—Mo1—N2—C8	−13.5 (3)	C10—C9—C14—O4	180.0 (3)
O1—Mo1—N2—C8	179.2 (4)	C8—C9—C14—O4	−3.2 (6)
O7—Mo1—N2—C8	−98.1 (3)	C10—C9—C14—C13	−0.3 (5)
O6—Mo1—N2—N1	−87.7 (3)	C8—C9—C14—C13	176.4 (3)
O5—Mo1—N2—N1	71.1 (4)	C20—N3—C16—C17	−0.9 (6)
O4—Mo1—N2—N1	173.2 (3)	N3—C16—C17—C18	−0.4 (6)
O1—Mo1—N2—N1	6.0 (2)	C16—C17—C18—C19	2.1 (6)
O7—Mo1—N2—N1	88.7 (2)	C16—C17—C18—C23	−178.4 (4)
C6—C1—C2—C3	1.4 (6)	C17—C18—C19—C20	−2.6 (6)
C7—C1—C2—C3	−177.8 (4)	C23—C18—C19—C20	178.0 (4)
C1—C2—C3—C4	−1.8 (6)	C16—N3—C20—C19	0.4 (7)
C2—C3—C4—O3	179.2 (4)	C18—C19—C20—N3	1.4 (8)
C2—C3—C4—C5	1.1 (7)	C25—N4—C21—C22	−0.3 (6)
O3—C4—C5—C6	−178.0 (4)	N4—C21—C22—C23	1.0 (6)
C3—C4—C5—C6	0.0 (7)	C21—C22—C23—C24	−0.8 (6)
C4—C5—C6—O2	177.9 (4)	C21—C22—C23—C18	178.6 (4)
C4—C5—C6—C1	−0.4 (6)	C19—C18—C23—C24	37.6 (6)
C2—C1—C6—O2	−178.5 (4)	C17—C18—C23—C24	−141.8 (4)
C7—C1—C6—O2	0.7 (6)	C19—C18—C23—C22	−141.7 (4)
C2—C1—C6—C5	−0.2 (6)	C17—C18—C23—C22	38.9 (6)
C7—C1—C6—C5	178.9 (4)	C22—C23—C24—C25	−0.1 (6)
N2—N1—C7—O1	−0.5 (5)	C18—C23—C24—C25	−179.4 (4)
N2—N1—C7—C1	−178.2 (3)	C21—N4—C25—C24	−0.6 (6)
Mo1—O1—C7—N1	6.3 (5)	C23—C24—C25—N4	0.8 (6)
Mo1—O1—C7—C1	−175.9 (3)		

### Hydrogen-bond geometry (Å, °)

**Table:**

*D*—H···*A*	*D*—H	H···*A*	*D*···*A*	*D*—H···*A*
O2—H2···N1	0.84 (1)	1.85 (3)	2.600 (4)	147 (6)
O3—H3···N3	0.84 (1)	1.92 (2)	2.741 (5)	166 (6)
O7—H7···N4^i^	0.84 (1)	1.84 (1)	2.679 (4)	175 (6)

Symmetry codes: (i) −*x*+1/2, *y*+1/2, −*z*+1/2.
